# Revealing the hidden burden: wastewater-based epidemiology for underreported and emerging infectious diseases in communities

**DOI:** 10.1007/s10661-026-15464-1

**Published:** 2026-05-19

**Authors:** Justin C. Greaves, Roberto A. Rodriguez

**Affiliations:** Department of Environmental and Occupational Health, School of Public Health, Innovation Center, Indiana University, 2719 E 10 Street, Bloomington, IN 47408 USA

**Keywords:** Wastewater-based epidemiology, Wastewater surveillance, Infectious diseases, Underreported, Emerging pathogens, Metagenomics

## Abstract

Wastewater-based epidemiology (WBE) has become a transformative tool for infectious disease surveillance, providing population-level insights that complement and extend traditional case-based reporting. This review examines the expanding role of WBE in identifying and characterizing underreported, novel, and emerging human pathogens. Evidence reveals that wastewater analysis consistently detects enteric, respiratory, and neglected pathogens that are often missed by clinical systems, thereby revealing the hidden burden of infection within communities. Sequencing-based studies have identified numerous novel and divergent human viruses, highlighting the extensive diversity of the human virome. The frequent co-detection of multiple viral taxa also suggests that interactions and co-infections may influence viral evolution, disease manifestation, and transmission. Despite methodological challenges in quantification and biological validation, WBE has proven capable of detecting both known and novel pathogens before they are clinically recognized. Future developments in long-read sequencing, bioinformatics, and global data integration will enhance the precision and scope of wastewater genomics, positioning it as a central element of early-warning and One Health surveillance frameworks. By illuminating the unseen spectrum of infectious agents, WBE bridges environmental and clinical domains, offering a scalable and equitable strategy for global pathogen discovery and public health preparedness.

## Introduction

Effective public health surveillance forms the cornerstone of disease prevention, control, and preparedness (Lee et al., 2011). Yet, despite significant advances in diagnostics, data systems, and epidemiological modeling, most surveillance frameworks still capture only a fraction of infections occurring within communities (Gibbons et al., 2014; Wolken et al., 2023). Large portions of transmission remain invisible because many individuals are never tested, cases go unreported, or infections are not clinically recognized (Gibbons et al., 2014; Pérez-Reche et al., 2021; Wolken et al., 2023). Underreporting, defined as the failure to capture all true cases of disease within surveillance systems, is one of the most persistent challenges in public health. It occurs when infected individuals do not seek medical care, are misdiagnosed, or when confirmed cases are not accurately recorded in national or regional databases (Albani et al., 2021). Underreporting distorts the understanding of disease burden and transmission dynamics. Its consequences are substantial, including missed outbreaks, delayed interventions, inefficient allocation of healthcare resources, and the perpetuation of health inequities. Diseases that cause mild or nonspecific symptoms, occur in marginalized populations, or rely on limited diagnostic infrastructure are particularly prone to underreporting (Albani et al., 2021; Gibbons et al., 2014; Pérez-Reche et al., 2021; Wolken et al., 2023). As a result, public health policy is frequently based on incomplete data that underestimates both the magnitude and distribution of infection within communities (Gibbons et al., 2014).

Addressing underreported diseases is essential for accurate risk assessment and equitable health protection. Hidden transmission sustains endemic circulation, enables silent spread across populations, and can lead to outbreaks that appear to emerge unexpectedly (Grubaugh et al., 2019; Shaikh et al., 2023). Chronic underreporting also conceals the proper distribution of disease burden, reinforcing social and spatial inequities in exposure and access to care (Meadows et al., 2022; Whittaker et al., 2021). Furthermore, many emerging and re-emerging pathogens, including zoonotic agents, may circulate undetected in human populations for extended periods before large-scale outbreaks occur (Berrian et al., 2024). Without mechanisms to detect these invisible trends, opportunities for early intervention are easily lost. Enhancing surveillance for underreported diseases is therefore critical for strengthening public health resilience and achieving global health security.

Wastewater surveillance offers a unique solution to these challenges by providing a biological and population-level means of detecting hidden infections (Mao et al., 2020; Thompson et al., 2020). Infected individuals shed pathogens, nucleic acids, or metabolic biomarkers through excreta that enter the sewage network (Rioux et al., 2023). These materials persist in wastewater long enough to be collected, concentrated, and analyzed (Rioux et al., 2023). Because wastewater reflects input from the entire connected population, it captures both symptomatic and asymptomatic infections, independent of healthcare access or testing behavior (Mao et al., 2020; Thompson et al., 2020). As a result, WBE can identify ongoing community transmission even when clinical case reports are minimal or absent (Sims & Kasprzyk-Hordern, 2020). The method is also scalable, anonymous, and adaptable to a wide range of pathogens, making it suitable for both high-resource and resource-limited settings (Sims & Kasprzyk-Hordern, 2020).

Beyond detection of known pathogens, WBE also holds potential for the discovery of previously unrecognized or emerging diseases (Grassly et al., 2025). Advances in metagenomics, sequencing, and bioinformatics have expanded WBE beyond targeted assays to untargeted community surveillance (Grimm et al., 2025). These approaches can reveal novel microbial signatures, track antimicrobial resistance genes, and identify shifts in the microbial ecology of urban environments (Davis et al., 2023; Grimm et al., 2025). Consequently, wastewater serves as an integrative archive of community health, offering insight into both recognized and emerging threats (Davis et al., 2023; Grimm et al., 2025).

This review examines the role of wastewater surveillance as a transformative tool for identifying and characterizing underreported diseases. Unlike prior reviews that primarily focus on individual pathogens or methodological advances, this work uniquely synthesizes how wastewater-based epidemiology reveals systematic underreporting across diverse pathogen classes while also enabling the detection of unmonitored and emerging infections. The paper begins by exploring how WBE complements traditional surveillance by detecting underreported infections at the community level and pathogens that are not well monitored through conventional methods. Key case studies are presented in which wastewater monitoring has revealed previously unrecognized circulation of enteric bacteria, viruses, and other neglected diseases. The review then discusses novel viruses discovered through wastewater sequencing and their implications for understanding hidden viral diversity in human populations. Finally, the review concludes with future directions for integrating WBE into global health infrastructure, with particular attention to genomic innovations, standardization, and equitable surveillance capacity. By linking underreporting, pathogen discovery, and wastewater genomics within a One Health framework, this review examines the broader role of WBE as both a surveillance and discovery platform for improving global public health preparedness.

## Evidence of underreporting in cases revealed by wastewater surveillance

**Fig. 1:**
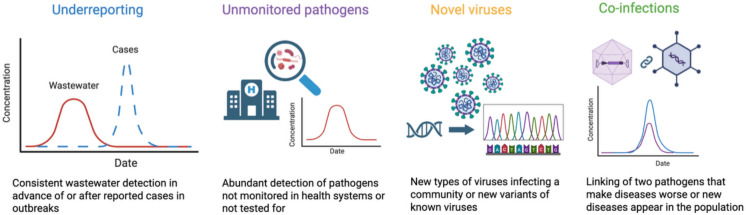
Various components of underreporting revealed by wastewater surveillance

Wastewater-based epidemiology (WBE) has generated extensive empirical evidence that infectious diseases are systematically underreported through traditional surveillance systems (Kilaru et al., 2022; Mao et al., 2020). Across a range of pathogen classes, wastewater surveillance has provided direct confirmation that official case data often capture only a small fraction of true infections, with some studies suggesting that reported cases may underestimate true infection rates by several-fold depending on the pathogen and setting (Gibbons et al., 2014; Huang et al., 2022; Levy et al., 2023; Wolken et al., 2023). While underreporting of well-known emerging pathogens has been the focus of many wastewater surveillance efforts, other aspects of underreporting include monitoring diseases not monitored by traditional surveillance systems, discovering novel viruses, and understanding co-infections between pathogens (Fig. 1). Figure 1 provides a conceptual overview of how wastewater surveillance captures multiple, often overlooked dimensions of infectious disease dynamics. This illustrates the ability of WBE to move beyond case-based reporting by integrating signals of hidden transmission, unmonitored pathogens, and pathogen interactions at the population level. The following sections summarize key findings from emerging known enteric and respiratory pathogens, illustrating the extent to which wastewater reveals underreported disease burdens or an early warning in communities.

### Enteric pathogens

Enteric viruses and bacteria provide some of the clearest evidence that wastewater surveillance can reveal substantial underreporting in traditional case-based systems (Table 1). Because these pathogens are shed in feces regardless of symptom severity, wastewater captures both symptomatic and asymptomatic infections, offering a sensitive population-level signal. Norovirus exemplifies this capacity as wastewater analyses in Europe, North America, and Asia consistently detect viral RNA year-round, even when few or no clinical cases are reported (Ammerman et al., 2024; Huang et al., 2022; Kazama et al., 2016). Quantitative comparisons indicate infection rates several-fold higher than those suggested by surveillance data, reflecting the high proportion of mild or asymptomatic infections that never reach healthcare systems (Guo et al., 2022). In many regions, wastewater concentrations rise 1 to 2 weeks before clinical case spikes, indicating the value of WBE for early warning and detection of otherwise silent community transmission (Ammerman et al., 2024; Huang et al., 2022; Kazama et al., 2016). Similar findings have been documented for hepatitis A virus (HAV), which often appears in wastewater weeks before reported cases, revealing cryptic circulation in presumed non-endemic areas and providing evidence of viral reintroduction (Zulli et al., 2024). Rotaviruses show parallel patterns: despite declining clinical reports following vaccination campaigns, viral genomes remain detectable in wastewater, suggesting continued low-level transmission among unvaccinated or subclinically infected individuals (Chan et al., 2025). Enteroviruses, including poliovirus, remain a classic example of WBE’s value in uncovering unrecognized transmission. Environmental detections in countries without paralytic cases, such as Israel and the UK, have prompted targeted immunization campaigns that successfully prevented outbreaks (ECDC, 2023; UKHSA, 2025). Bacterial and protozoan pathogens show comparable trends. Salmonella, Campylobacter, Vibrio, and Shigella DNA are routinely detected in sewage, even when reported incidence is low, whereas Cryptosporidium is often detected in the absence of clinical confirmation (Goldblum et al., 2024; Xiao et al., 2022a, b).

**Table 1:** Selected studies showing underreporting of various viral pathogens

Pathogen/focus	Region (sampling years)	Key observation (wastewater vs clinical cases)	Reference
Norovirus (temporal dynamics in sewage)	Japan (sampling through 2013–2016; paper 2016/2017)	Sewage norovirus concentrations tracked genotype dynamics and showed persistent detection, often when clinical case surveillance reported low activity; peaks in sewage sometimes led clinical reports	Kazama et al. (2016)
Norovirus (multi-site WBE)	USA (multi-plant, 2020 s; paper 2024)	Wastewater HuNoV GII measurements indicated ongoing community circulation and produced earlier signals than syndromic/outbreak reports; sewage showed prevalence outside reported case surges	Ammerman et al. (2024)
Hepatitis A virus (HAV)	USA (2023–2024)	HAV RNA detected across wastewater plants and in some cases present prior to and persisting after official outbreak notifications, indicating silent/ongoing transmission not fully captured by case reports	Zulli et al. (2024)
Rotavirus (post-vaccine detection in sewage)	USA (studies through 2016–2020)	Rotavirus genomes remained detectable in wastewater after the introduction of routine vaccination, even as clinical case counts fell, suggesting continued low-level circulation	Chan et al. (2025)
Human astroviruses (novel clades MLB/VA)	USA, South Korea, Netherlands (2015–2021)	Novel astrovirus lineages were found more frequently in wastewater than in routine clinical detection; wastewater suggested broader circulation, including periods with few clinical reports	Hata et al. (2015)
Respiratory syncytial virus (RSV)	USA, Canada, UK (2021–2023)	RSV RNA in wastewater rose before, or more strongly than, clinical sentinel data in several studies, providing earlier indications of seasonal activity and continued circulation when case reports were low	Wolfe et al. (2022)
SARS-CoV-2	Global (2020–2022)	Wastewater SARS-CoV-2 levels often anticipated rises in reported cases by days to weeks and remained detectable when testing and case reporting declined, validating WBE as an early and resilient indicator	Peccia et al. (2020)
Adeno-associated virus 2 (AAV-2) and adenovirus 41	UK (2022–2023)	Retrospective wastewater sequencing detected AAV-2 circulation before clinical recognition of pediatric hepatitis clusters, supporting environmental evidence of pre-existing circulation. The study in the US showed a high prevalence of these pathogens in wastewater, with no clinical cases reported	Martin et al. (2023); Rodriguez et al. (2024)

Collectively, these findings demonstrate a consistent pattern in which wastewater surveillance detects sustained and often early circulation of enteric pathogens that remain substantially under-represented in clinical reporting systems. Across geographic regions and pathogen types, WBE provides a more complete picture of infection dynamics, capturing both symptomatic and asymptomatic transmission and highlighting its value as a sensitive, population-level indicator of enteric disease burden.

### Respiratory pathogens

The COVID-19 pandemic provided the most striking demonstration where wastewater concentrations of SARS-CoV-2 consistently rose several days to multiple weeks before increases in clinically confirmed cases, hospitalizations, or positive tests, according to several previous studies (Galani et al., 2022; Peccia et al., 2020; Xiao et al., 2022a, b). Even as widespread at-home testing and reporting fatigue reduced clinical data completeness in later phases of the pandemic, SARS-CoV-2 RNA continued to be detected reliably in sewage, revealing ongoing transmission that case reports failed to capture (Brighton et al., 2024). Across diverse geographic and socioeconomic settings, these patterns demonstrated the scalability and robustness of wastewater surveillance as a population-level monitoring tool.

Beyond its role in tracking SARS-CoV-2, the pandemic provided a large-scale validation of wastewater-based epidemiology as a core public health surveillance system. In multiple countries, WBE functioned independently of clinical testing infrastructure, maintained surveillance during periods of reduced reporting, and provided early-warning signals that informed public health interventions. These findings suggest that WBE can serve not only as a complementary tool but as a resilient backbone for population-level infectious disease monitoring.

Similar patterns have been observed for influenza viruses, where wastewater detection frequently precedes or exceeds surveillance based on sentinel healthcare systems (Wolfe et al., 2022). In several US cities, influenza A and B RNA concentrations correlated strongly with community infection but revealed circulation weeks before official reporting spikes (Wolfe et al., 2022). Respiratory syncytial virus (RSV) and human metapneumovirus (HMPV) have also been detected in wastewater before or independent of documented outbreaks, underscoring how WBE captures community transmission among both symptomatic and asymptomatic carriers (Allen et al., 2024).

These findings indicate that wastewater surveillance can provide a consistent and early indicator of respiratory pathogen dynamics across diverse settings, although uncertainties in shedding variability, environmental factors, and quantitative interpretation remain. Continued integration of WBE with clinical and epidemiological data, along with advances in standardization and modeling, will be essential for strengthening its role in global infectious disease surveillance and pandemic preparedness.

### Limitations of WBE

Though there may be many benefits to WBE in terms of underreported diseases, WBE is not immune to bias. Detection efficiency depends on pathogen-specific shedding dynamics, environmental stability, and analytical sensitivity (Singh et al., 2024). Pathogens that are excreted intermittently, shed at low titers, or rapidly degrade in sewage may be systematically under-represented, leading to false negatives or underestimated prevalence (Kilaru et al., 2022; Rioux et al., 2023). For example, SARS-CoV-2 exhibits significant inter-individual variability in fecal viral shedding: some infected individuals release high viral loads, while others shed little or none (Arts Peter et al., 2023; Lavania et al., 2022). This variability can cause temporal gaps in detection, particularly when sampling frequency or concentration methods are suboptimal. Similarly, hepatitis E virus (HEV) and mycobacterium tuberculosis complex (MTBC) are known to shed intermittently or at low levels into wastewater, complicating quantitative interpretation even when transmission is ongoing (Dimeglio et al., 2025; Mtetwa et al., 2022). Moreover, nucleic acid decay rates vary across pathogens; enveloped viruses such as SARS-CoV-2 or influenza degrade faster than non-enveloped enteric viruses, introducing additional uncertainty (Ahmed et al., 2020). These factors mean that, just as clinical data can underestimate infection, wastewater data may under-detect pathogens with unfavorable shedding or stability characteristics. Ongoing advances in sample concentration, molecular recovery efficiency, and modeling of shedding variability are therefore essential to improve the accuracy and interpretability of WBE results.

In addition to these biological and analytical challenges, several broader limitations affect the interpretation and application of wastewater-based epidemiology. Methodological variability across studies, including differences in sampling strategies, concentration techniques, and molecular assays, can limit comparability and hinder the development of standardized frameworks. Linking wastewater pathogen concentrations to clinical case numbers remains complex due to variability in individual shedding rates, population size, and sewer system dynamics. Environmental factors such as temperature, precipitation, and wastewater composition can further influence pathogen persistence and detection. Finally, while WBE is typically conducted at aggregated population levels, ethical and privacy considerations may arise when surveillance is conducted at smaller spatial scales, emphasizing the need for careful implementation and governance.

## Hidden pathogens and diseases not monitored by traditional surveillance methods

The large-scale deployment of wastewater surveillance during the COVID-19 pandemic demonstrated that population-level pathogen monitoring is feasible at a global scale. These advances have enabled the expansion of WBE beyond well-known pathogens to a broader spectrum of underreported and emerging infectious agents. Many infectious agents continue to circulate silently within human populations with little clinical or public health attention (Shaikh et al., 2023; Vallejo et al., 2022). These “under-the-radar” pathogens are often mild, difficult to culture, or lack standardized diagnostic assays, which leads to substantial underrecognition in clinical data (Alsharksi et al., 2024; Liu et al., 2023). Yet, their consistent detection in wastewater indicates that they are far more prevalent than conventional surveillance systems suggest (Alsharksi et al., 2024; Liu et al., 2023). Importantly, these hidden agents are not insignificant. Persistent low-level circulation may contribute to chronic disease burden, influence co-infection dynamics, and provide opportunities for viral recombination, antimicrobial resistance, or future emergence events (Grassly et al., 2025). Ignoring such pathogens risks missing early signals of shifts in microbial ecology that could have broader health implications. Understanding their presence and prevalence through wastewater-based epidemiology (WBE) is therefore essential for building a more complete picture of population health and strengthening outbreak preparedness. The following sections summarize emerging evidence for underreported or overlooked pathogens identified through WBE, including lesser-studied enteric and respiratory viruses, oral and skin-associated microorganisms, and other agents that have received minimal clinical or research attention.

### Enteric pathogens

Human bocavirus (HBoV), first identified in 2005, is an enteric and respiratory virus that remains poorly characterized epidemiologically (Allander et al., 2005). Genogroup 1 is responsible for respiratory illness, whereas genogroups 2–4 are responsible for gastrointestinal illnesses (Kapoor et al., 2010). Clinical testing for HBoV is uncommon, and its symptoms, typically mild respiratory or gastrointestinal illness, are often attributed to other agents (Chiu et al., 2024; Jartti et al., 2012). Additionally, genogroups 2–4 remain uncultivable in the lab, making them challenging to study. Wastewater surveillance has repeatedly detected HBoV DNA in samples from diverse geographic regions, including Europe, Asia, and the Americas (Alam et al., 2015; Cheng et al., 2008; Kumthip et al., 2021; La Rosa et al., 2018; Malta et al., 2020). In many of these studies, HBoV detection frequency and viral loads were comparable to those of well-established enteric viruses such as norovirus or adenovirus. Persistent detection throughout the year, including in the absence of reported outbreaks, suggests continuous community circulation. The high prevalence of HBoV in sewage indicates widespread, largely unrecognized infection as well as the potential of wastewater data to illuminate its epidemiology and seasonal dynamics. These observations suggest that HBoV is likely circulating more widely than clinical data indicate, reflecting the limited routine testing and nonspecific symptoms associated with infection. Wastewater surveillance therefore offers a useful population-level perspective, helping to clarify its true prevalence and epidemiological patterns.

Aichi virus, a member of the Picornaviridae family, is another underreported enteric pathogen (Ambert-Balay et al., 2008). It has been linked to foodborne and waterborne gastroenteritis, yet clinical testing remains rare due to the lack of routine diagnostic assays (Rivadulla & Romalde, 2020). Multiple wastewater studies across Japan, Europe, and the Middle East have detected AiV RNA consistently over time, with some showing higher prevalence than other enteric viruses that receive more attention (Alcalá et al., 2010; Burutarán et al., 2016; Di Martino et al., 2013; Kitajima et al., 2011; Lodder et al., 2013; Sdiri-Loulizi et al., 2010). The virus’s persistence in wastewater samples indicates that infections are common, though seldom reported (Chuchaona et al., 2017). Wastewater surveillance has the ability to reveal the silent prevalence of foodborne pathogens that remain virtually invisible to clinical surveillance.

Other enteric agents of emerging interest beyond HBoV and AiV include human astroviruses, sapoviruses, and adeno-associated viruses. These pathogens cause sporadic illness but are rarely included in routine surveillance systems (Di Bartolo et al., 2013; Hata et al., 2015). Astroviruses and sapoviruses, for example, are frequently identified in sewage in the absence of corresponding case reports, suggesting widespread under detection in children and immunocompromised individuals (Di Bartolo et al., 2013; Hata et al., 2015). Similarly, adeno-associated viruses were found in connection with enteric adenoviruses to cause acute hepatitis of unknown origin in children recently, pointing to unrecognized community circulation (Rodriguez et al., 2024). These detections reveal a diverse range of enteric pathogens whose epidemiology can only be adequately captured through WBE.

### Respiratory pathogens

While wastewater monitoring of respiratory pathogens expanded dramatically during the COVID-19 pandemic, several respiratory viruses continue to receive minimal attention in clinical or environmental surveillance. One study has identified human metapneumovirus (hMPV), parainfluenza viruses, and seasonal coronaviruses (e.g., OC43, 229E, NL63) in wastewater at measurable concentrations (Boehm et al., 2023; Chan & Boehm, 2025). Despite causing significant respiratory illness, these viruses are often underreported because testing is typically limited to hospitalized cases or sentinel surveillance networks. Wastewater data indicate that their circulation is more widespread and usually overlaps with influenza and SARS-CoV-2 activity in possible co-infections (Boehm et al., 2023; Chan & Boehm, 2025). The detection of these pathogens in sewage indicates that wastewater monitoring can complement respiratory disease surveillance by capturing broader viral diversity and identifying off-season or multi-pathogen transmission events that might otherwise go unnoticed.

### Dental pathogens

Dental and oral-associated bacteria have received limited attention in public health surveillance despite their significant role in global disease burden (Wu et al., 2025). Periodontal and dental infections are among the most common chronic conditions worldwide, yet their microbial epidemiology is rarely captured outside of clinical dentistry. Wastewater surveillance provides an indirect but valuable window into oral health at the community level, revealing patterns of microbial presence and potential population-scale trends in oral disease (Birch et al., 2025a, b). One study showed key oral pathogens, such as *Streptococcus mutans* and *Porphyromonas gingivalis*, have been identified in municipal wastewater and sludge, reflecting widespread colonization and active disease within communities (Birch et al., 2025a, b). *Streptococcus mutans*, the primary etiological agent of dental caries, was particularly notable for its high concentrations and detections in wastewater. *S. mutans* plays a central role in biofilm formation and acid production, leading to enamel demineralization (Caufield, 2000; Forssten et al., 2010; Lemos et al., 2019). The identification of both bacteria in wastewater reflects the potential of WBE to serve as a population-scale indicator of oral microbial ecology and periodontal disease prevalence.

### Skin-associated pathogens

Wastewater studies have also revealed the presence of skin-associated microorganisms, including opportunistic pathogens that can cause infections in immunocompromised individuals. Studies have focused on surveillance for emerging well-known pathogens, such as mpox virus, but organisms, such as *Staphylococcus aureus* and *Pseudomonas aeruginosa*, are also commonly identified in sewage samples (Amirsoleimani et al., 2019; Schwartz et al., 2006). Their detection likely reflects skin cell shedding, bathing, and hygiene activities that contribute microbial biomass to wastewater systems. While these pathogens are part of normal human microbiota, the consistent detection of virulent or antibiotic-resistant strains suggests that WBE could be leveraged to monitor community-level trends in skin and soft-tissue infections (Amirsoleimani et al., 2019; Schwartz et al., 2006). In particular, tracking methicillin-resistant *Staphylococcus aureus* (MRSA) DNA in wastewater may provide early warnings of emerging resistance hotspots that are not yet apparent from hospital surveillance data (Goldstein et al., 2012). Additionally, other common pathogens shed through the skin, such as Molluscum contagiosum virus and *Streptococcus pyrogenes*, are missing in traditional surveillance, but have also been detected through recent wastewater surveillance efforts (Birch et al., 2025; Kuo et al., 2023). These observations illustrate a key dimension of underreporting in infectious disease surveillance. Many skin-associated pathogens are not systematically monitored at the population level, as infections are often mild, treated empirically, or only documented in clinical settings without broader epidemiological tracking. Wastewater-based epidemiology therefore provides a unique opportunity to capture community-level trends in these infections, including the emergence of antibiotic-resistant strains, offering insights that are largely inaccessible through traditional surveillance systems.

### Other hidden pathogens

**Fig. 2:**
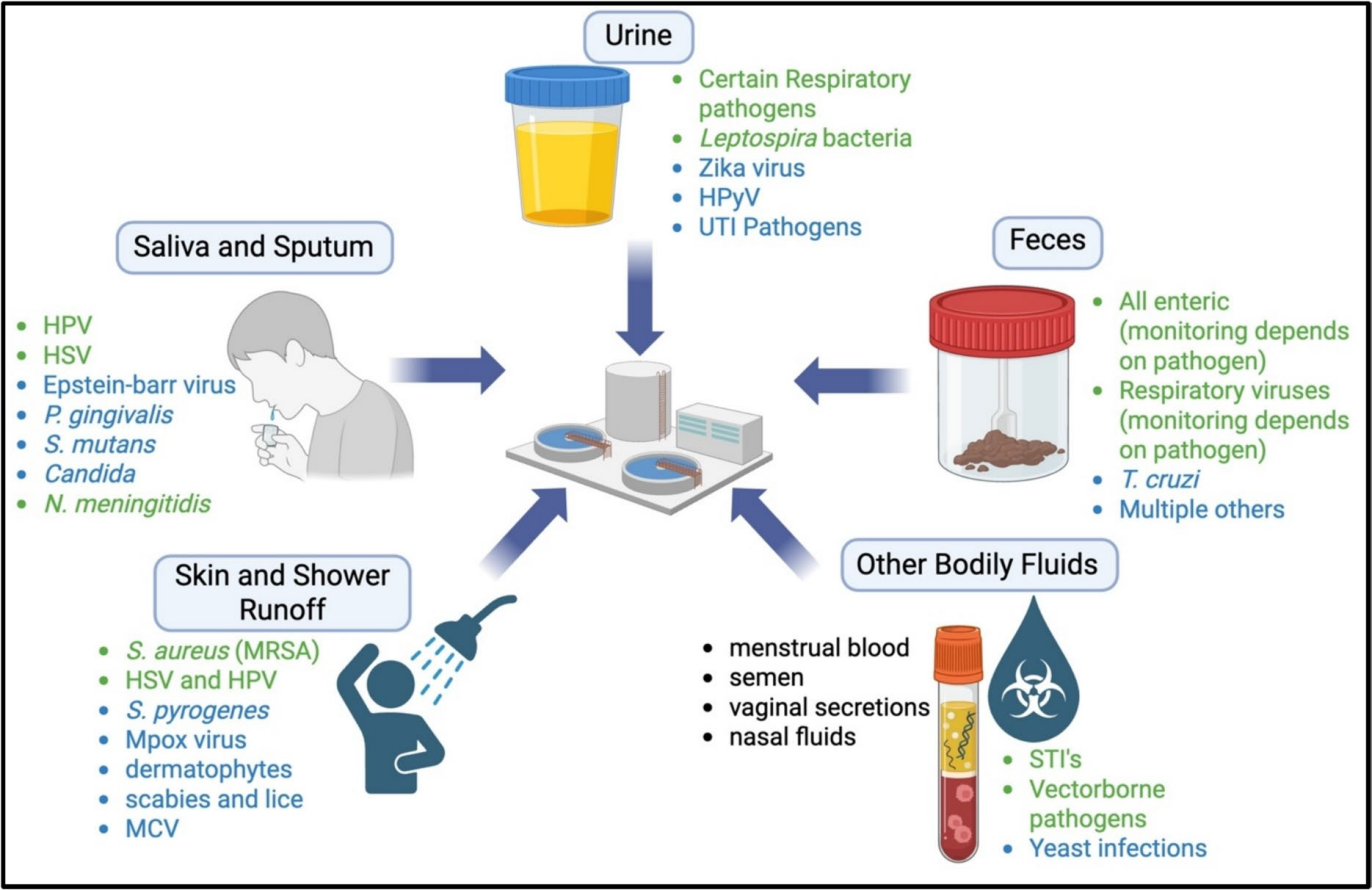
Various pathogen shedding routes into the wastewater treatment plant and the various undermonitored pathogens (blue) within each route

Beyond enteric, respiratory, and skin-associated agents, wastewater analyses have uncovered multiple additional pathogens that remain largely unreported in traditional public health surveillance (Fig. 2). Figure 2 highlights the diverse biological pathways through which pathogens enter wastewater systems, including fecal, respiratory, skin, and oral shedding. This integrative view emphasizes the unique strength of wastewater surveillance in capturing a wide range of underreported and under-monitored pathogens simultaneously, reinforcing its role as a comprehensive tool for population-level infectious disease monitoring. Torque teno virus (TTV), a small single-stranded DNA virus of uncertain pathogenicity, is detected globally in sewage and is increasingly considered a potential indicator of immune function at the population scale (Charest et al., 2015; Gore et al., 2023). Likewise, human papillomaviruses (HPV) and herpesviruses have been identified in wastewater, providing new opportunities to explore their epidemiology, which goes beyond the traditional sexually transmitted diseases that are regularly monitored (Alshehri et al., 2025; Giesbrecht Shayna et al., 2025). Additionally, numerous diseases worldwide have affected populations in certain countries in endemics, such as neglected tropical diseases, and recent data has distinctly shown the benefits of WBE for the pathogens that cause these diseases (Monteiro et al., 2025; Morin & Alfahl, 2025). Collectively, these findings highlight a broader category of underreported and under-monitored pathogens that fall outside conventional surveillance priorities. Because many of these agents are not routinely tested for or are associated with asymptomatic or chronic infections, their true prevalence remains poorly understood. Wastewater surveillance uniquely enables the detection and characterization of these pathogens at the population level, demonstrating its added value as a comprehensive tool for uncovering hidden dimensions of infectious disease burden.

## Discovery of new pathogens through wastewater surveillance and sequencing

The emergence of high-throughput sequencing has transformed wastewater-based epidemiology (WBE) into a powerful tool for viral discovery. Wastewater represents a composite biological sample of entire communities, containing nucleic acids shed by infected individuals through feces, urine, saliva, and other excreta. Metagenomic and metatranscriptomic sequencing of these samples allows researchers to characterize the complete viral diversity present in human populations, independent of clinical testing or prior knowledge of specific pathogens (Mechikoff et al., 2024). This approach has revealed an extraordinary range of both known and previously unrecognized viruses, many of which have uncertain pathogenic potential (Delmas et al., 2019). The ability of wastewater sequencing to capture the genetic signatures of circulating, emerging, and novel pathogens establishes it as an early-warning and discovery system for infectious disease surveillance.

### Novel viruses

Although no human pathogenic virus has yet been discovered de novo solely from wastewater data, wastewater metagenomics has enabled the identification of numerous previously unknown bacteriophages and diverse small DNA and RNA viruses that infect non-human hosts. These discoveries which include many different bacteriophages and many CRESS-DNA lineages demonstrate the power of environmental sequencing to reveal viral taxa that were entirely invisible to traditional clinical surveillance (Rosario et al., 2019; Strange et al., 2021). As sequencing technologies, bioinformatic tools, and reference databases continue to advance, the capacity to detect genuinely novel human-infectious viruses in wastewater may soon become feasible, providing a valuable complement to fecal and clinical sampling.

Wastewater studies have already expanded the known diversity of multiple human-associated virus families by identifying new variants, divergent genotypes, and recombinant forms circulating silently within communities. For example, picobirnaviruses (PBVs) which were discovered through human stool in 1988 were revisited through next-generation sequencing in wastewater in recent years, and data has uncovered extensive genomic diversity and newly emerging lineages (Delmas et al., 2019). Several wastewater studies have reported novel PBV sequences with less than 60% nucleotide identity to known reference strains, implying multiple unclassified species within the Picobirnaviridae (Guajardo-Leiva et al., 2020; Kashnikov et al., 2020; Zhang et al., 2015). PBVs were also identified in wastewater from both urban and rural areas, regardless of reported gastrointestinal illness, supporting the hypothesis that these double-stranded RNA viruses are widespread, largely asymptomatic human infections that may occasionally contribute to disease in immunocompromised hosts (Guajardo-Leiva et al., 2020; Kashnikov et al., 2020; Zhang et al., 2015).

Since 2016, metagenomic analyses of wastewater have also revealed significant diversity within established enteric virus families, such as picornavirus. Many strains within picornavirus have been detected in sewage, and the diversity expanded (Bibby & Peccia, 2013; Guajardo-Leiva et al., 2020; Han et al., 2010; Stöcker et al., 2012). In 2018–2019, environmental sequencing expanded understanding of astrovirus diversity (Hata et al., 2015). Beyond the classical human astrovirus types 1–8, novel lineages, including Astrovirus MLB and Astrovirus VA/HMO, were consistently detected in wastewater from the USA and Asia (Hata et al., 2015, 2018). These variants, characterized by distinct open reading frame (ORF) arrangements and capsid structures, have since been linked in clinical studies to encephalitis and meningitis in children and immunocompromised patients (Bami et al., 2022; Cordey et al., 2016). Finally, ongoing metagenomic work since 2020 has uncovered a diverse set of small circular DNA viruses, including CRESS-DNA and nanoviruses, that occur consistently in human wastewater worldwide (Rosario et al., 2019). Although their pathogenicity remains uncertain, their persistent detection in urban sewage indicates they are integral to the human-associated virome (Rosario et al., 2019). Their identification underscores how much of the human viral diversity remains unexplored even in well-sampled environments.

### Other novel microbial pathogens

Wastewater metagenomics has not only advanced viral discovery but has also revealed a remarkable diversity of bacterial and eukaryotic genomes, many of which are poorly characterized yet potentially pathogenic. Predicting new pathogenic bacterial and eukaryotic genomes is significantly more difficult, but previous studies have shown the power of wastewater surveillance for this in terms of antimicrobial resistance. Large-scale metagenomic analyses of urban sewage across multiple continents have revealed thousands of previously unclassified bacterial metagenome-assembled genomes (MAGs), many belonging to genera that include opportunistic or emerging pathogens such as Pseudomonas, Acinetobacter, Mycobacterium, and Enterococcus (Hendriksen et al., 2019; Yan et al., 2025). These findings suggest that wastewater serves as a reservoir and conduit for globally circulating bacterial lineages, including those harboring antimicrobial resistance (AMR) genes and virulence factors. Similarly, eukaryotic sequencing of wastewater, particularly targeting fungi, protists, and other microeukaryotes, has uncovered previously unrecognized diversity within clinically important genera such as Candida, Aspergillus, Cryptococcus, and Naegleria (Persyn et al., 2022). Some of these lineages contain genetic determinants of stress tolerance or antifungal resistance, underscoring the potential for wastewater-based metagenomics to track emerging or environmentally persistent eukaryotic pathogens (Persyn et al., 2022).

## Relationship between pathogens and co-infection

Emerging evidence also suggests that various types of pathogens may not act in isolation. Certain pathogens can enhance the infection of other pathogens. For instance, several studies have shown *Salmonella enterica* and other pathogenic bacteria can enhance enteric virus infection in the body and stability, such as poliovirus and coxsackievirus (Dhalech Adeeba et al., 2021; Greaves et al., 2025). This relationship is an example of the potential beneficial effect of certain pathogens. Other relationships have shown a conflicting effect where one pathogen limits another so that its infections may be enhanced. An example of this is human rhinovirus limiting the infection of certain types of respiratory viruses, such as SARS-CoV-2, in order for its infection to proliferate (Dee et al., 2021). There is a potential for these relationships, especially for pathogens that coinfect, to be revealed through disease trends in wastewater-based surveillance where all these pathogens would be shed and be present in wastewater.

Wastewater sequencing often detects multiple viral groups co-circulating within the same community samples, implying that co-infection and viral interactions could be common in the human gut (Kumblathan et al., 2024; Lu et al., 2025). Furthermore, co-infections between adenoviruses and AAV are essential for AAV replication and may modulate disease outcomes, as mentioned before in recent pediatric hepatitis clusters (Rodriguez et al., 2024). Similarly, concurrent shedding of enteroviruses, parechoviruses, and astroviruses has been observed, raising the possibility of synergistic or competitive interactions that influence infection severity or transmission dynamics (Kapusinszky et al., 2012). Such relationships may also facilitate genetic recombination or reassortment, contributing to the evolution of novel viral variants. Understanding these ecological and functional linkages among enteric viruses will be critical for interpreting wastewater-derived signals and assessing their relevance to human disease emergence.

## Conclusions

Wastewater-based epidemiology (WBE) has evolved from a niche environmental monitoring tool into a cornerstone of modern infectious disease surveillance. Across this review, evidence shows that wastewater analysis consistently reveals infections and pathogens that traditional clinical systems overlook, from underreported and unmonitored enteric diseases to respiratory and emerging viruses. The application of high-throughput sequencing has further transformed WBE into a potential discovery platform, uncovering a remarkable diversity of human-associated viruses that remain absent from routine diagnostics and public health reporting. Looking forward, wastewater surveillance holds immense potential for early warning and underreported pathogen discovery within a One Health framework. Continued refinement of long-read sequencing, viral enrichment, and metagenomic approaches will improve genome completeness and enable real-time tracking of viral evolution. Global coordination of wastewater genomic data could create a continuous, population-scale observatory for emerging infectious threats even when they go unnoticed in traditional surveillance.

## Data Availability

The data that support the review is available within the article.
